# *FOXO1* gene involvement in a non-rhabdomyosarcomatous neoplasm

**DOI:** 10.1007/s00428-021-03026-4

**Published:** 2021-01-28

**Authors:** Simon Haefliger, Muriel Genevay, Michel Bihl, Romina Marone, Daniel Baumhoer, Michael Papaloizos, Matthias S. Matter, Beata Bode-Lesniewska

**Affiliations:** 1 Institute of Medical Genetics and Pathology, University Hospital of Basel, University of Basel, Schönbeinstrasse 40, CH-4031 Basel, Switzerland; 2 Institute of Pathology Dianapath, Geneva, Switzerland; 3 Department of Biomedicine, University Hospital of Basel, University of Basel, Basel, Switzerland; 4 Bone Tumor Reference Center at the Institute of Medical Genetics and Pathology, University Hospital of Basel, University of Basel, Basel, Switzerland; 5 Center for Hand Surgery and Therapy, Geneva, Switzerland; 6 Institute of Pathology Enge, Zürich, Switzerland

**Keywords:** *OGT*, *FOXO1*, Myoepithelioma, Rhabdomyosarcoma

## Abstract

Myoepithelial neoplasms of soft tissue are rare tumors with clinical, morphological, immunohistochemical, and genetic heterogeneity. The morphological spectrum of these tumors is broad, and the diagnosis often requires immunostaining to confirm myoepithelial differentiation. Rarely, tumors show a morphology that is typical for myoepithelial neoplasms, while the immunophenotype fails to confirm myoepithelial differentiation. For such lesions, the term “myoepithelioma-like” tumor was introduced. Recently, two cases of myoepithelioma-like tumors of the hands and one case of the foot were described with previously never reported *OGT-FOXO* gene fusions. Here, we report a 50-year-old woman, with a myoepithelial-like tumor localized in the soft tissue of the forearm and carrying a *OGT-FOXO1* fusion gene. Our findings extend the spectrum of mesenchymal tumors involving members of the FOXO family of transcription factors and point to the existence of a family of soft tissue tumors that carry the gene fusion of the OGT-FOXO family.

## Introduction

Myoepithelial neoplasms of soft tissue and skin are rare tumors with clinical, morphological, immunohistochemical, and genetic heterogeneity [1]. They arise most commonly on the extremities and limb girdles, affecting all age groups with a peak in young to middle-aged adults [1, 2]. Myoepithelial neoplasms are classified as myoepitheliomas, myoepithelial carcinomas, and mixed tumors. Mixed tumors contain additionally ductal differentiation. The morphological spectrum of myoepithelial neoplasms comprises lobular or nodular growth patterns with epithelioid or spindled cells organized in cords, nests, single-cell or trabecular patterns, and a chondromyxoid to hyaline background [1, 2]. Immunohistochemically, the majority of myoepithelial neoplasms express at least one epithelial marker, e.g., cytokeratins or epithelial membrane antigen (EMA) and S100 protein. Furthermore, the variable expression of myogenic markers (SMA, desmin), GFAP, and p63 can be observed. Molecular studies have led to a better characterization of myoepithelial neoplasms at the genetic level but also demonstrated considerable heterogeneity. *EWSR1* gene rearrangements with various fusion partners were found besides fusions with *FUS*, *SRF-2E2F1*, and *PLAG1* [1, 3, 4].

Histopathological diagnosis remains challenging due to the microscopic, immunohistochemical, and genetic heterogeneity of myoepithelial tumors, which is further complicated by tumors which share the morphology but not the typical immunohistochemistry. As a consequence, the term “myoepithelial-like” tumor has been used in cases where the microscopic features were not supported by a confirming immunophenotype. Myoepithelioma-like tumors have been found especially in the vulvar region, where they all showed a loss of *SMARCB* expression [5]. Thus, molecular findings suggested different subtypes. Indeed, Lee et al. recently reported two cases of myoepithelioma-like tumors with a previously unknown *OGT-FOXO3* gene fusion [2]. Similarly, Yorozu et al. described one case of a myoepithelioma-like tumor of the foot with a previously unknown *OGT-FOXO1* gene fusion [6]. *FOXO1* and *FOXO3* gene belong to the forkhead box O (FOXO) subclass of transcription factors, including also the *FOXO4* gene. *FOXO3* and *FOXO4* have been reported as fusion partners in leukemias, whereas *FOXO1* is a well-known fusion partner in alveolar rhabdomyosarcoma (ARMS), as *PAX3-FOXO1* and *PAX7-FOXO1* [7].

Here, we describe a unique myoepithelial-like tumor of the forearm of a 50-year-old woman carrying a *OGT-FOXO1* gene fusion.

## Materials and methods

### Material

Specimen was formalin-fixed and paraffin-embedded (FFPE) and sections were cut for conventional histology before staining with H&E. Immunohistochemistry (IHC) was performed on 2-μm-thick paraffin sections, using the Ventana Benchmark XT automated staining system (Ventana Medical Systems, Tucson, AZ). The following antibodies were used: ALK1 (D5F3), CD34 (QBEnd/10), Desmin (DE-R-11), ERG (5B7), EMA (E29), INI-1 (MRQ27), Ki67 (30-9), p63 (4A4), panCK (AE1/AE3), S100 (polyclonal), SOX10 (SP267), and STAT6 (EP325) all from Roche/Ventana (Tucson, AZ) and SMA (BS66, Biosite, San Diego, CA).

The study was conducted according to the standard regulations of the local ethical committee (BASEC 2018-01922). Written informed consent was obtained from the patient for publication.

### RNA isolation and sequencing

Total RNA was extracted by using the Maxwell® RSC RNA FFPE Kit (Promega, Madison, WI). Libraries were prepared using a customized RNA-based NGS panel (Archer™ FusionPlex, ArcherDx, Inc., Boulder, CO), containing 51 genes known to be translocated in tumors [8]. Two hundred fifty nanograms of RNA was used for generating NGS libraries, which were loaded to an Ion 540™ chip (Thermo Fisher Scientific, Waltham, MA) for sequencing. Raw data were processed automatically on the Torrent Server™ v5.10 and aligned to the hg19 reference genome. Bam files were uploaded into the Archer data analysis pipeline (Archer™ analysis software version 6.0).

### Fluorescence in situ hybridization

For fluorescence in situ hybridization a *FOXO1* SPEC Dual Color Break Apart Probe (ZytoVision, Bremerhaven, Germany; Cat-No. Z-2139-50) was used for the detection of specific translocations involving the chromosomal region 13q14.11 harboring the *FOXO1*. The orange fluorochrome direct-labeled probe hybridizes distal; the green fluorochrome direct-labeled probe hybridizes proximal to the breakpoint region of the *FOXO1* gene. For visualization of the probes, 4-μm-thick FFPE slides were deparaffinized, pretreated, and hybridized overnight.

### Reverse transcription polymerase chain reaction

For confirmation of RNA sequencing data, RNA was retrotranscribed to cDNA and subjected to reverse transcription polymerase chain reaction (RT-PCR). The primers used were forward primer 1 *OGT*: GACATAGCTGTGAAGCTGGGA, reverse primer 1 *FOXO1*: TCGGCTTCGGCTCTTAGCAA, reverse primer 2 *FOXO1*: CTTGCTGTGTAGGGACAGAT, and reverse primer 3 *FOXO1*: CAGAGTGAGCCGTTTGTCCG). RT-PCR was performed using SuperScript IV First-Strand Synthesis System (Invitrogen, Carlsbad, CA) and Phusion Green Hot Start II High-Fidelity DNA Polymerase (Thermo Scientific, Waltham, MA) at an annealing temperature of 60 °C for 35 cycles. The PCR products were then analyzed by gel electrophoresis and Sanger sequencing.

## Results

### Clinical findings, morphological and immunohistochemical analysis

**Fig. 1:**
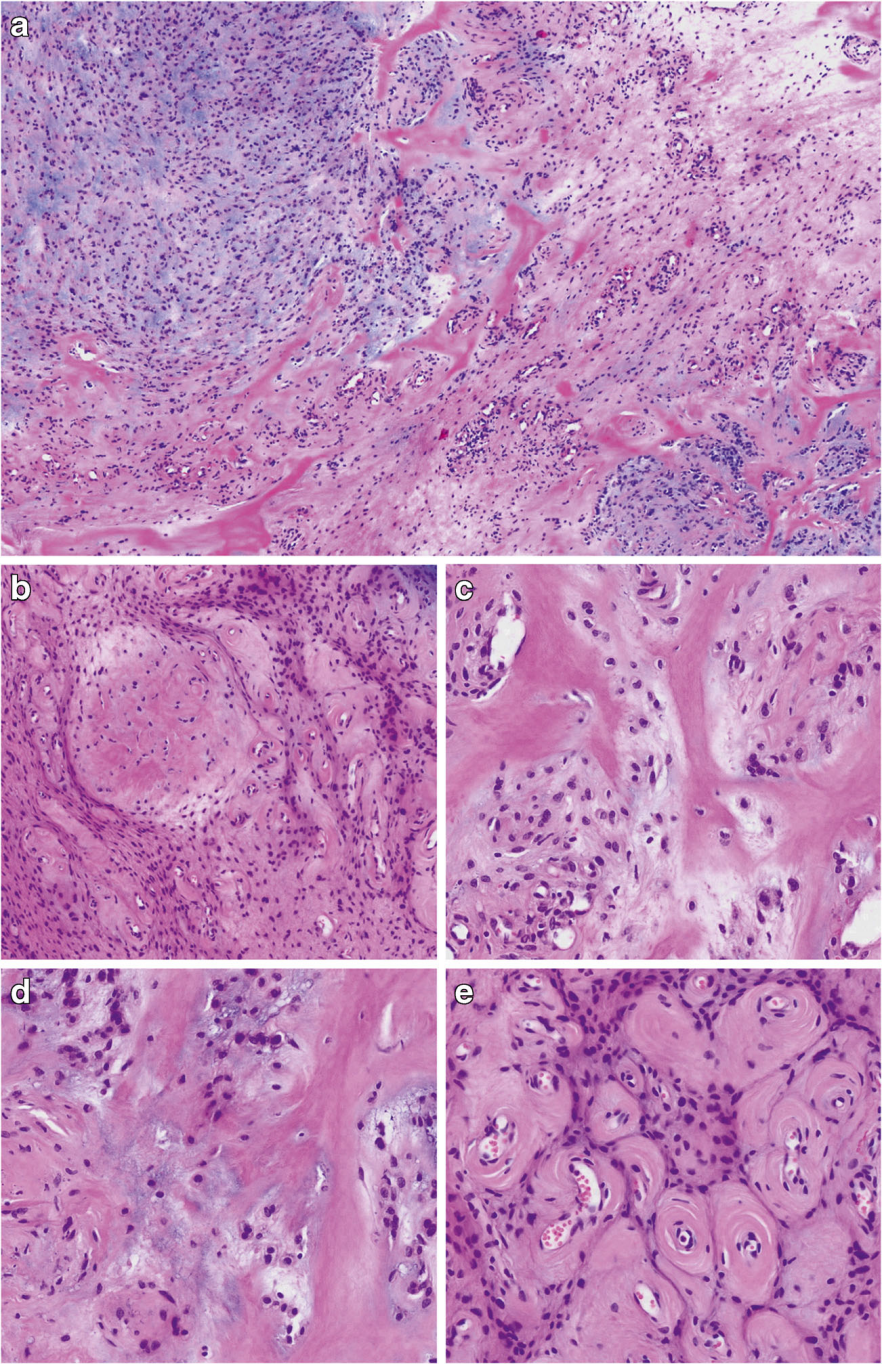
Histomorphological analysis. **a** At low-power view, the tumor tissue shows a heterogeneous appearance with areas consisting of chondromyxoid matrix and others with fibrosis (×25, H&E) **b** Tumor cells are arranged in loose cords, single cells, or a reticular pattern (×50, H&E). **c**–**d** The tumor stroma consists mainly of broad hyaline collagen (C, ×100, H&E) and areas with deposition of chondromyxoid material (D, ×100, H&E). **e** At the periphery of the tumor, numerous small blood vessels with concentric perivascular hyalinization are found (×100, HE)

**Fig. 2:**
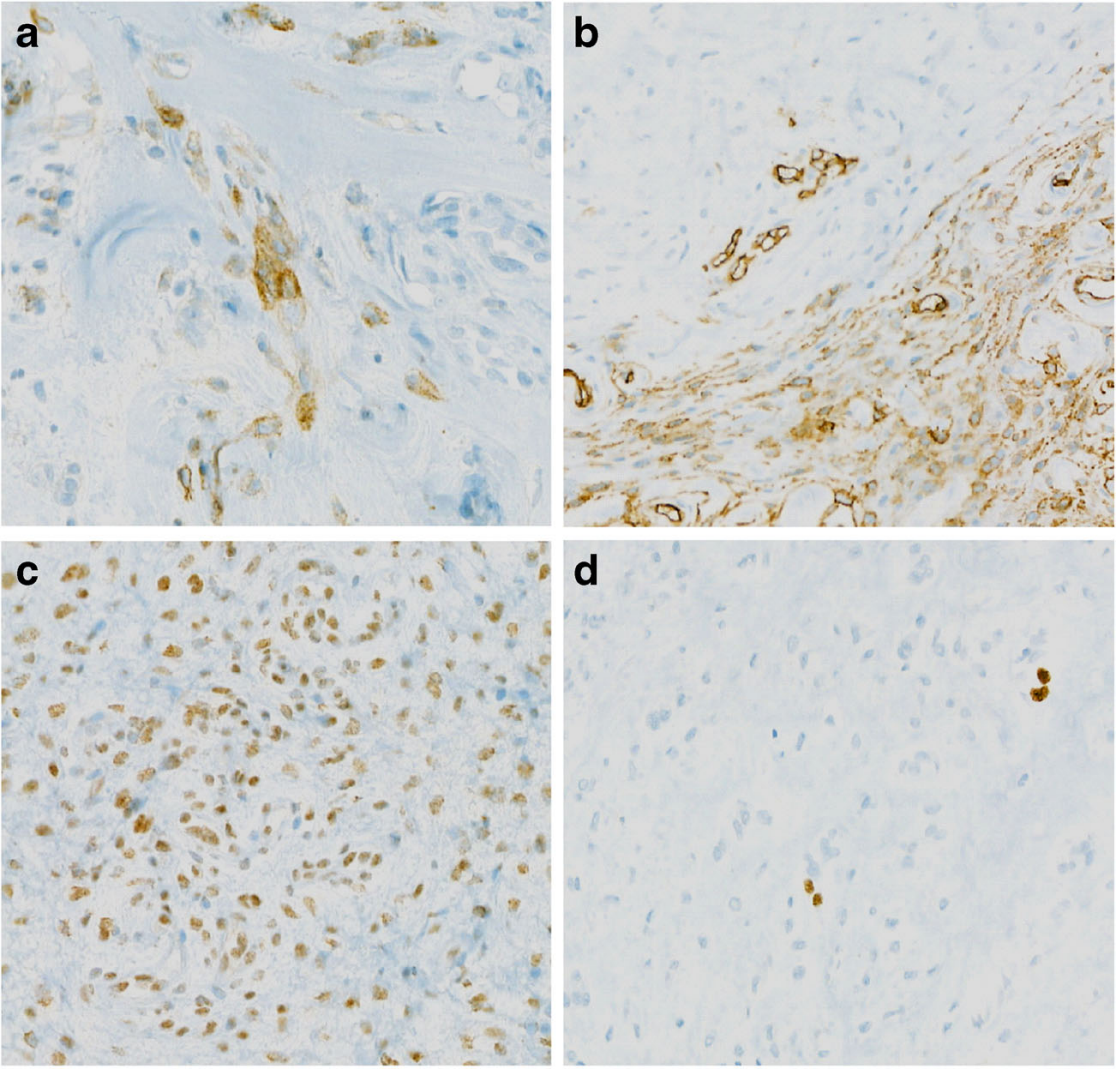
Representative immunohistochemical findings: **a** EMA expression in a few tumor cells (×200). **b** Focal positivity for CD34 (×100). **c** Retained nuclear expression of INI1 protein (×200). **d** Low proliferation index (Ki-67-staining) (×200)

A 50-year-old woman of African origin presented with a slowly growing, painless, and firm subcutaneous mass on the dorsal aspect of the left mid-forearm, which measured two centimeters in diameter. The lesion was well defined, mobile with respect to the skin, and slightly adherent to the underlying tissue. No pre-operative imaging was performed, and presumptive diagnosis was that of a lipoma. At surgery, a whitish, well-demarcated tumor was found, which was attached to the aponeurosis of the left extensor digitorum. Microscopically, the tumor consisted of a lobulated, well-defined soft tissue tumor, composed of cells arranged in cords, nests, and single cells, or disposed in a reticular/trabecular pattern (Fig. 1a–b). The background consisted of a sclerosing and chondromyxoid stroma with dense eosinophilic pericellular and perivascular collagen deposition (Fig. 1c–d). The cells showed a monotonous appearance with uniformly rounded nuclei with dense chromatin without nucleoli. A prominent vasculature composed of small-sized, often slightly dilated vessels with pronounced perivascular collagen deposition was noted, mainly in the periphery of the tumor (Fig. 1e). Mitotic activity was low, and areas of necrosis were not observed. Immunohistochemical analysis failed to show convincing myoepithelial differentiation as the tumor cells were only focally positive for EMA and CD34 (Fig. 2a–b). The reactions against AE1/AE3, S100, SOX10, SMA, Desmin, ALK1, ERG, p63, and STAT6 were all negative. The nuclear expression of INI1 protein was retained, and the proliferation index (Ki-67) was below 5% (Fig. 2c–d). Since the precise histopathological subclassification of the tumor could not be done, the sample was submitted for NGS analysis.

### *OGT-FOXO1* fusion

**Fig. 3:**
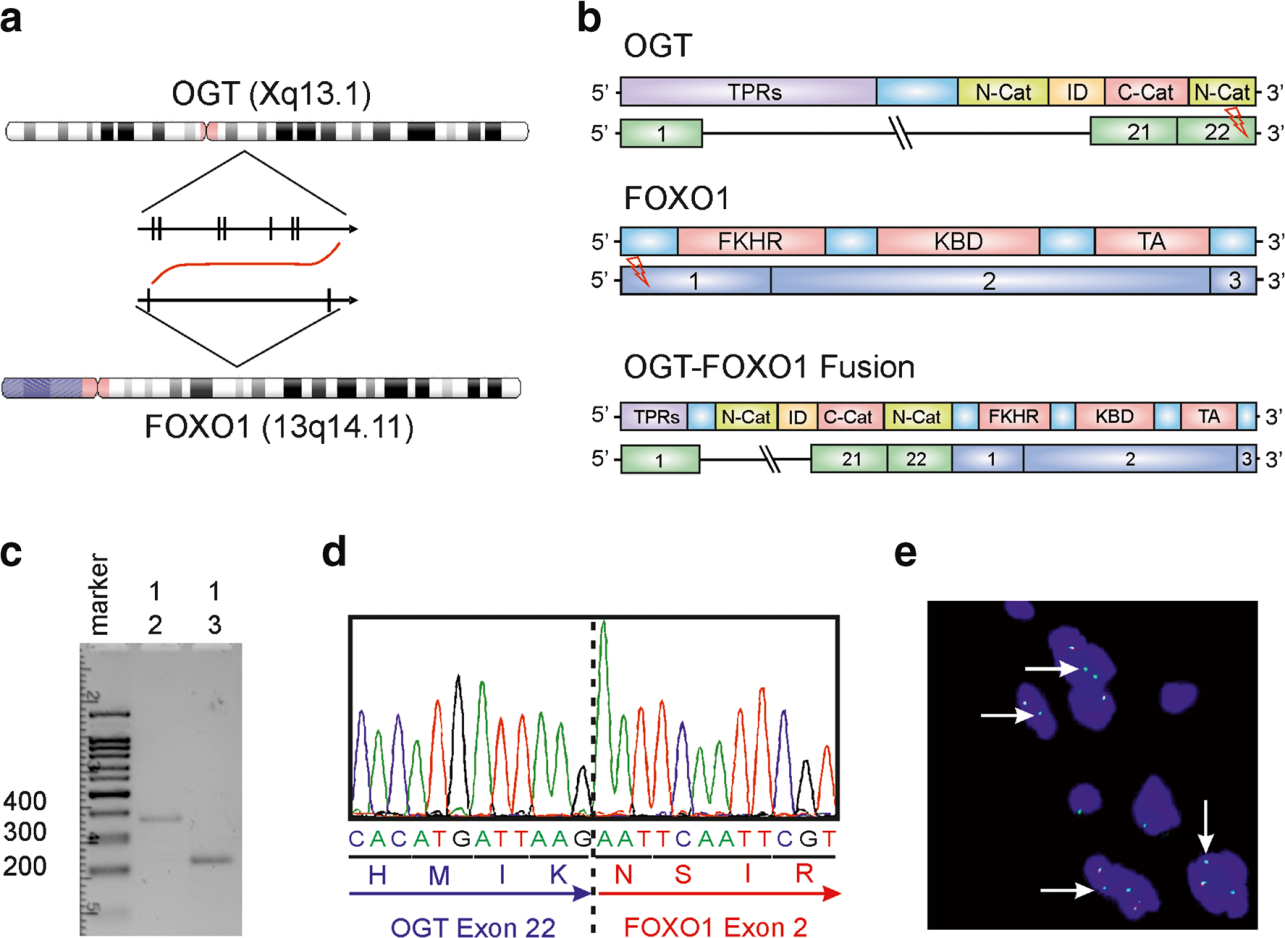
Molecular analysis of *OGT-FOXO1* fusion. **a** Chromosomal location and orientation of *OGT* and *FOXO1*. **b** Schematic image of the exons and domain composition of the partner genes and fusion gene. The breakpoints were found in exon 22 of *OGT* and exon 1 of *FOXO1*. **c**–**d** RT-PCR was performed using a forward primer from *OGT* (primer 1) and two different reverse primers from *FOXO1* (primers 2 and 3). Each primer combination revealed a band of a fusion product between 200 and 400 bp(C). Sanger sequencing of the fusion product confirmed the same sequence as detected by NGS (D). **e** Fluorescence in situ hybridization. FISH analysis was performed using a *FOXO1* Break Apart Probe and revealed an unusual pattern with one fused signal and one single green signal (white arrow)

For molecular analysis, we performed RNA sequencing using the Archer Anchored Multiplex (AMP™) PCR technology. Interestingly, we detected a novel *OGT-FOXO1* gene fusion. The breakpoints were within exon 22 (the last exon) of *OGT* on chromosome X and exon 2 of *FOXO1* on chromosome 13, resulting in a fusion transcript containing most of the coding regions of both partner genes (Fig. 3a–b). Next, we performed RT-PCR using primers on both genes, which confirmed the in-frame fusion between *OGT* and *FOXO1* by gel electrophoresis and Sanger sequencing (Fig. 3c–d). Finally, we performed FISH analysis (Fig. 3e) with a *FOXO1* Break Apart Probe. This analysis revealed an atypical signal pattern with one fused signal and one single green signal in approximately 50% of the tumor cells, a phenomenon which is well known and usually corresponds to a deletion of parts of the gene in the presence of a fusion [9].

Finally, the diagnosis of a myoepithelial-like tumor of the soft tissue was made, carrying a *OGT-FOXO1* gene fusion. On follow-up examination after 8 months, there were no signs of local recurrence or metastatic spread.

## Discussion

Here we describe a unique soft tissue tumor of the forearm of a middle-aged female patient, with a morphology that showed the most similarities with a myoepithelioma. Because the immunophenotype did not unequivocally support this diagnosis, we classified the lesion as a myoepithelioma-like soft tissue tumor without features of malignancy. Interestingly, the tumor showed striking morphological and immunohistochemical similarities with three myoepithelioma-like soft tissue tumors recently described by Lee et al. and Yorozu et al. [2, 6]. Both cases reported by Lee et al. harbored a *OGT-FOXO3* protein fusion, whereas Yorozu et al. also detected a *OGT-FOXO1* fusion [2, 6].

*FOXO1* is a transcription factor belonging to the forkhead box O-class subfamily, which also includes *FOXO3*, *FOXO4*, and *FOXO6* [10]. FOXO proteins are implicated in a broad range of cellular functions, including cellular differentiation, apoptosis, and cell proliferation [11, 12]. FOXO1 proteins are usually regarded as tumor suppressors [11]. However, recent data challenged the sole tumor suppressive role of FOXO1 proteins and indicated a more complex role with tumor promoting roles in some tumors [12]. Interestingly, Karanian et al. [13] reported a *SRF-FOXO1* gene fusion in a low-grade rhabdomyosarcoma in a 10-month-old girl. The involvement of the *FOXO1* gene in the pathogenesis of a non-rhabdomyosarcoma soft tissue tumor without histological features of malignancy has only been reported previously by Yorozu et al. [6]. The other partner of the *OGT-FOXO1* is the O-GlcNAc transferase *(OGT)* gene, which is an enzyme catalyzing protein glycosylation and playing a crucial role in gene transcription, protein stabilization, and degradation [14]. Recent studies suggested that *OGT* activity promoted tumor growth [15]. The function of the *OGT-FOXO1* fusion transcript presented here remains to be elucidated. However, the modification of *FOXO1* activity through increased glycosylation but also increased *OGT* activity might represent a possible oncogenic mechanism.

In summary, we report a case of myoepithelioma-like tumor of the hand with a *OGT-FOXO1* gene fusion, supporting previous evidence that myoepithelioma-like tumors with gene fusions involving *OGT* and the *FOXO* subfamily represent a distinct and novel tumor entity, whose biological behavior still has to be determined. We provide further evidence that *FOXO1* might be involved in the pathogenesis of non-rhabdomyosarcoma mesenchymal tumors.

## Data Availability

Not applicable.
